# Aerosol-spray diverse mesoporous metal oxides from metal nitrates

**DOI:** 10.1038/srep09923

**Published:** 2015-04-21

**Authors:** Long Kuai, Junxin Wang, Tian Ming, Caihong Fang, Zhenhua Sun, Baoyou Geng, Jianfang Wang

**Affiliations:** 1 College of Chemistry and Materials Science, The Key Laboratory of Functional Molecular Solids, Ministry of Education, Anhui Laboratory of Molecular-Based Materials, Center for Nano Science and Technology, Anhui Normal University, Wuhu 241000, China; 2 Department of Physics, The Chinese University of Hong Kong, Shatin, Hong Kong SAR, China; 3 Shenyang National Laboratory for Materials Science, Institute of Metal Research, Chinese Academy of Sciences, Shenyang 110016, China

## Abstract

Transition metal oxides are widely used in solar cells, batteries, transistors, memories, transparent conductive electrodes, photocatalysts, gas sensors, supercapacitors, and smart windows. In many of these applications, large surface areas and pore volumes can enhance molecular adsorption, facilitate ion transfer, and increase interfacial areas; the formation of complex oxides (mixed, doped, multimetallic oxides and oxide-based hybrids) can alter electronic band structures, modify/enhance charge carrier concentrations/separation, and introduce desired functionalities. A general synthetic approach to diverse mesoporous metal oxides is therefore very attractive. Here we describe a powerful aerosol-spray method for synthesizing various mesoporous metal oxides from low-cost nitrate salts. During spray, thermal heating of precursor droplets drives solvent evaporation and induces surfactant-directed formation of mesostructures, nitrate decomposition and oxide cross-linking. Thirteen types of monometallic oxides and four groups of complex ones are successfully produced, with mesoporous iron oxide microspheres demonstrated for photocatalytic oxygen evolution and gas sensing with superior performances.

Transition metal oxides have applications in a wide range of technologies^1^,^2^,^3^,^4^,^5^,^6^,^7^,^8^,^9^,^10^. Many of their applications benefit from the formation of porous materials with large surface areas and pore volumes, as well as the formation of complex oxides, such as mixed, doped, multimetallic oxides and oxide-based hybrids. Continuous efforts have been made on the synthesis of mesoporous transition metal oxides since the 1990s owing to their wide uses in various important applications as mentioned above. Poly(alkylene oxide) block copolymers have predominantly been employed to direct the formation of mesoporous metal oxides through casting, film coating or aerosol spray. Two major types of metal-containing compounds have been utilized as metal oxide precursors. One is metal chlorides^11^,^12^,^13^,^14^,^15^, and the other is metal alkoxides^16^,^17^,^18^,^19^,^20^. Careful control of the hydrolysis and condensation rates of metal species in solutions has been found to be crucial for the formation of mesostructured oxides. Restrained hydrolysis and condensation have been realized through the inherent generation of strong hydrochloric acid for chloride precursors and the addition of strong acids for alkoxide precursors. Besides their separate uses, the two types of precursors are also often mixed together for better control of mesostructure formation and for the creation of mixed or multimetallic oxides^21^,^22^,^23^,^24^. The involvement of strong acids makes the synthesis highly corrosive and environment-unfriendly. In addition, metal alkoxides are generally expensive and unstable.

We recently reported on the use of Fe(NO_3_)_3_ and Ni(NO_3_)_2_ as oxide precursors to prepare amorphous mixed iron-nickel oxides at varying compositions through aerosol spray for efficient electrochemical oxygen evolution reaction^25^. Here we demonstrate the versatility and powerfulness of this cost-effective approach for the synthesis of a variety of mesoporous metal oxides ranging from monometallic to complex ones. The approach combines aerosol spray together with solvent evaporation-induced assembly. Low-cost metal nitrate salts are used as oxide precursors. The precursor solutions are composed only of metal nitrates, poly(alkylene oxide) block copolymer molecules, and ethanol. During the spray process, each droplet of the precursor solution acts as a microscale reactor. Solvent evaporation first induces mesostructure formation. The subsequent temperature increase after the solvent is completely evaporated causes the thermal decomposition of metal nitrates. This prevents precipitation and crystallization of oxides in the droplets and consequent phase separation between the inorganic and organic ingredients, a notably long-lasting problem in the synthesis of mesoporous transition metal oxides with chlorides and alkoxides as oxide precursors^11^,^12^,^13^,^14^,^15^,^16^,^17^,^18^,^19^,^20^,^21^,^22^,^23^,^24^. The separation of the mesostructure formation and the thermal decomposition of metal nitrates avoids the use of strong acids in the preparation. Our method is therefore easy to handle, and can potentially be upgraded for continuous and large-scale production of various mesoporous metal oxides. Thirteen types of monometallic oxides and four groups of complex ones have been successfully produced, with mesoporous iron oxide microspheres demonstrated for photocatalytic oxygen evolution and gas sensing with superior performances.

## Results and discussion

### Mesoporous monometallic metal oxides

**Figure 1:**
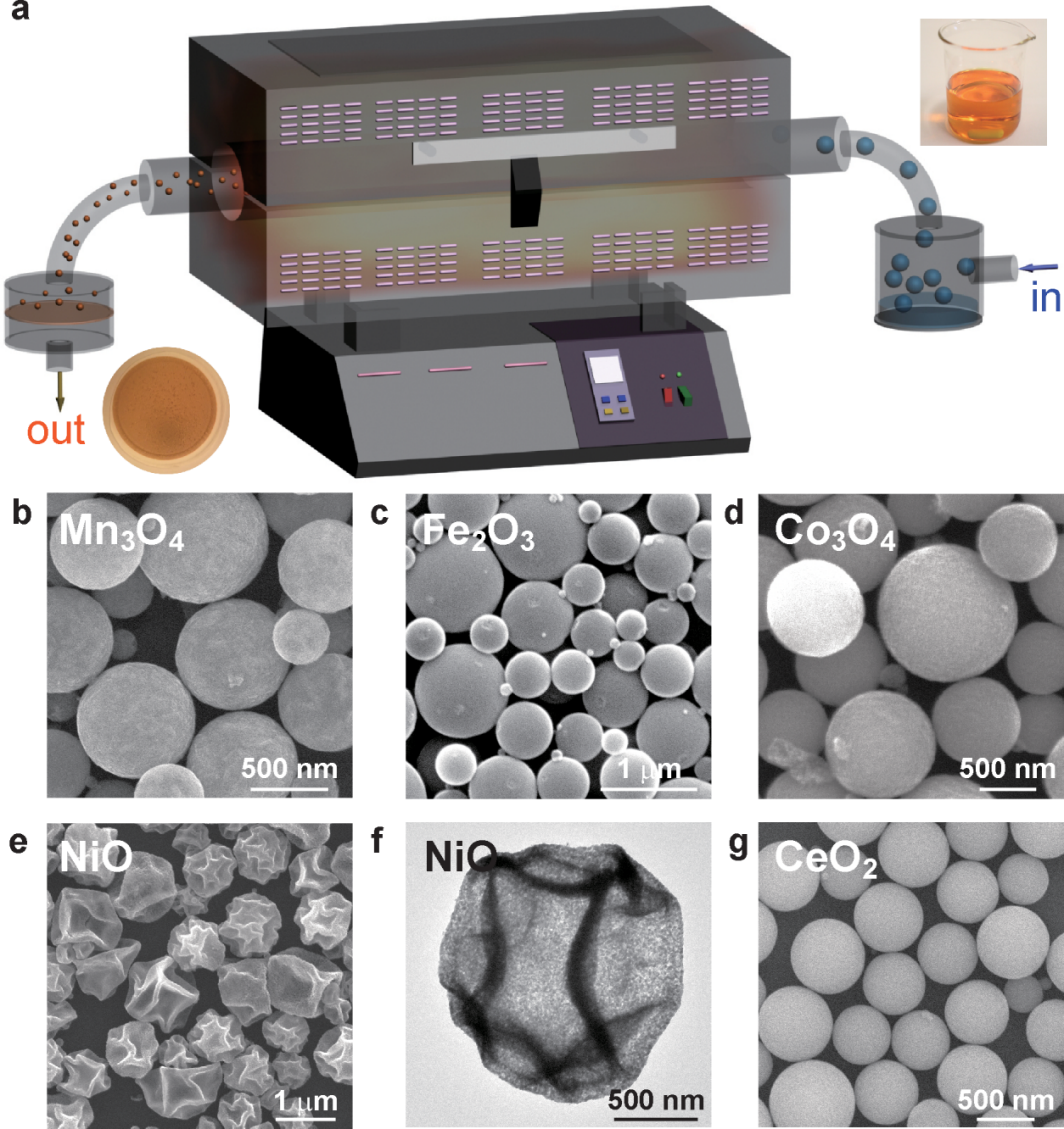
Aerosol spray system and representative monometallic oxide products. (a) Schematic illustrating the home-built setup and the processes associated with aerosol spray. The droplets of the precursor solution are carried into the furnace by nitrogen gas. Thermally-driven solvent evaporation and nitrate decomposition produce mesostructured oxide microspheres, which are collected with a filter. The top-right and bottom-left photographs show the precursor solution and the mesoporous microsphere product for Fe_2_O_3_, respectively. (b–e,g) SEM images of mesoporous monometallic Mn_3_O_4_, Fe_2_O_3_, Co_3_O_4_, NiO and CeO_2_ products. (f) TEM image of a single hollow crumpled NiO microsphere. The shown mesoporous oxide products were all calcined.

Aerosol spray was performed on a home-built system, which was composed of a household ultrasonic humidifier, a tube furnace, and a sample collector (Fig. 1a and Supplementary Fig. 1). The precursor solution was prepared from metal nitrate, poly(ethylene oxide)-poly(propylene oxide)-poly(ethylene oxide) triblock copolymer, and ethanol. It was nebulized with the humidifier. The generated mist was carried into the furnace with nitrogen. The furnace was maintained at a given temperature during spray. The residence time of the mist in the furnace under our spray conditions was 3–5 s. The production rate of mesoporous oxide products with our home-built system was estimated to be 0.1 g h^−1^.

We first present the mesoporous monometallic oxide products. A total of 13 types of monometallic oxides was successfully produced. They were characterized with scanning/transmission electron microscopy (SEM/TEM, Fig. 1b–g and Supplementary Fig. 2), nitrogen sorption (Supplementary Figs 3 and 4), and powder X-ray diffraction (XRD, Supplementary Fig. 5). In Table 1 are provided the Brunauer-Emmett-Teller surface areas and pore volumes determined from the nitrogen sorption measurements, together with the furnace temperatures used during the spray process and the thermal decomposition temperatures of the metal nitrate salts found in the literature (Supplementary Table S1). The mesoporous oxide products have a spherical shape, with diameters ranging from ~200 nm to ~2 μm, which are mainly determined by the droplet diameter and precursor concentration for a given material. For example, when 2 mmol of Al(NO_3_)_3_ or Fe(NO_3_)_3_ was used together with 0.25 g of the block copolymer surfactant, the average diameters of the obtained mesoporous Al_2_O_3_ and Fe_2_O_3_ spheres were 0.7 ± 0.3 μm and 0.8 ± 0.2 μm, respectively. When the concentrations of the nitrate and surfactant in the precursor solutions were reduced by half, the products remained to be spherical, with the corresponding average diameters becoming 0.5 ± 0.3 μm and 0.4 ± 0.3 μm. CuO microspheres are loosely packed. NiO and MgO microspheres are hollow, with folds appearing on their shells. The rest are densely packed. The loosely packed or hollow structures for the CuO, NiO and MgO products might result from the lower solubilities of the corresponding metal ions and thus their smaller diffusion rates in ethanol during the decomposition and condensation process in the furnace^26^. The specific surface areas range from ~20 m^2^ g^−1^ to ~200 m^2^ g^−1^, and the pore volumes range from ~0.05 cm^3^ g^−1^ to 0.5 cm^3^ g^−1^. The pores are generally disordered. Six types of mesoporous oxides, which are Fe_2_O_3_, CeO_2_, Al_2_O_3_, Cr_2_O_3_, Ga_2_O_3_ and ZrO_2_, display narrow Barrett-Joyner-Halenda pore size distributions, with their pore sizes centered in the range of ~5 nm to ~12 nm. The oxide products with narrow pore size distributions mostly have amorphous pore frameworks (Fe_2_O_3_, Al_2_O_3_, Ga_2_O_3_ and ZrO_2_), except CeO_2_ and Cr_2_O_3_, which have narrow pore size distributions as well as crystalline pore frameworks. The others exhibit broad pore size distributions, which are caused by crystalline pore frameworks, loosely packed or hollow interiors.

We found that it is essential to set the temperature of the furnace 200–300°C higher than the thermal decomposition temperature. If the furnace temperature is lower than ~300°C, the collected products are gel-like, suggesting that the solvent evaporation is incomplete. As the furnace temperature is increased, the products become dry. However, if the furnace temperature is less than ~200°C higher than the thermal decomposition temperature of the metal nitrate, the dry particles in the products fall apart by themselves. They are difficult to be processed further for characterization or use. Only if the furnace temperature is high enough, can the particles in the products be tight enough to maintain their integrity during further processing. During aerosol spray, each droplet acts as a microscale reactor. The spherical morphology of the product particles is determined by the shape of the liquid droplets. We surmise that in the droplets, solvated metal nitrate species interact mainly with the poly(ethylene oxide) part of the triblock copolymer molecules. Ethanol evaporation induces the formation of mesostructures, where the hydrophilic phase is composed of metal nitrate species and the poly(ethylene oxide) part of the copolymer, and the hydrophobic phase is composed of the poly(propylene oxide) part of the copolymer. If the furnace temperature is high enough, the temperature of the self-assembled microspheres will be further raised after ethanol is completely evaporated, which causes decomposition of metal nitrate species and subsequent condensation of the corresponding metal oxides. Our preparation method is also applicable for some metal oxides that do not have corresponding nitrate precursors but can be dissolved in ethanol, decomposed and condensed at raised temperatures. An example is WO_3_, for which H_2_WO_4_ was used as the precursor.

The mesoporous monometallic oxides can be post-treated to yield different phases with desired properties. For example, calcination of the product sprayed from Fe(NO_3_)_3_⋅9H_2_O at 400°C in air produced mesoporous Fe_2_O_3_ microspheres with an amorphous pore framework (Supplementary Fig. 5). Raising the calcination temperature to 500°C yielded mesoporous α-Fe_2_O_3_ microspheres (Supplementary Fig. 6). Thermally treatment of the α-Fe_2_O_3_ product at 400°C in a H_2_–N_2_ mixture gave Fe_3_O_4_. γ-Fe_2_O_3_ was obtained by thermally treating the Fe_3_O_4_ product at 400°C in air. Both of the Fe_3_O_4_ and γ-Fe_2_O_3_ products are ferromagnetic (Supplementary Fig. 7).

### Complex mesoporous metal oxides

Our spray approach is also capable of facilely producing complex mesoporous metal oxides beyond the monometallic ones, as demonstrated with doped, mixed, bimetallic oxides and oxide-based hybrids. Doping is a powerful means for adjusting the optical and electronic properties of metal oxide semiconductors. In our study, ZnO was taken as an example (Supplementary Fig. 8). Up to 10 mol% of Co, Mn, Ni, Cr and Fe could be readily doped in mesoporous ZnO, while the crystalline structure of the ZnO host framework remained unchanged. Such heavy doping led to the increases in the absorption intensity below the bandgap energy of ZnO over the visible and near-infrared regions. As the dopant concentration was increased, the absorption in the below-bandgap spectral region became intensified. When the Co concentration was increased from 0 mol% to 10 mol%, the diffraction peak at 2*θ* = 36.3° shifted to the high-angle region by only ~0.05°, because the ionic radius of Co is slightly smaller than that of Zn. This observation confirms the maintenance of the crystalline structure of the ZnO host framework upon doping.

**Figure 2:**
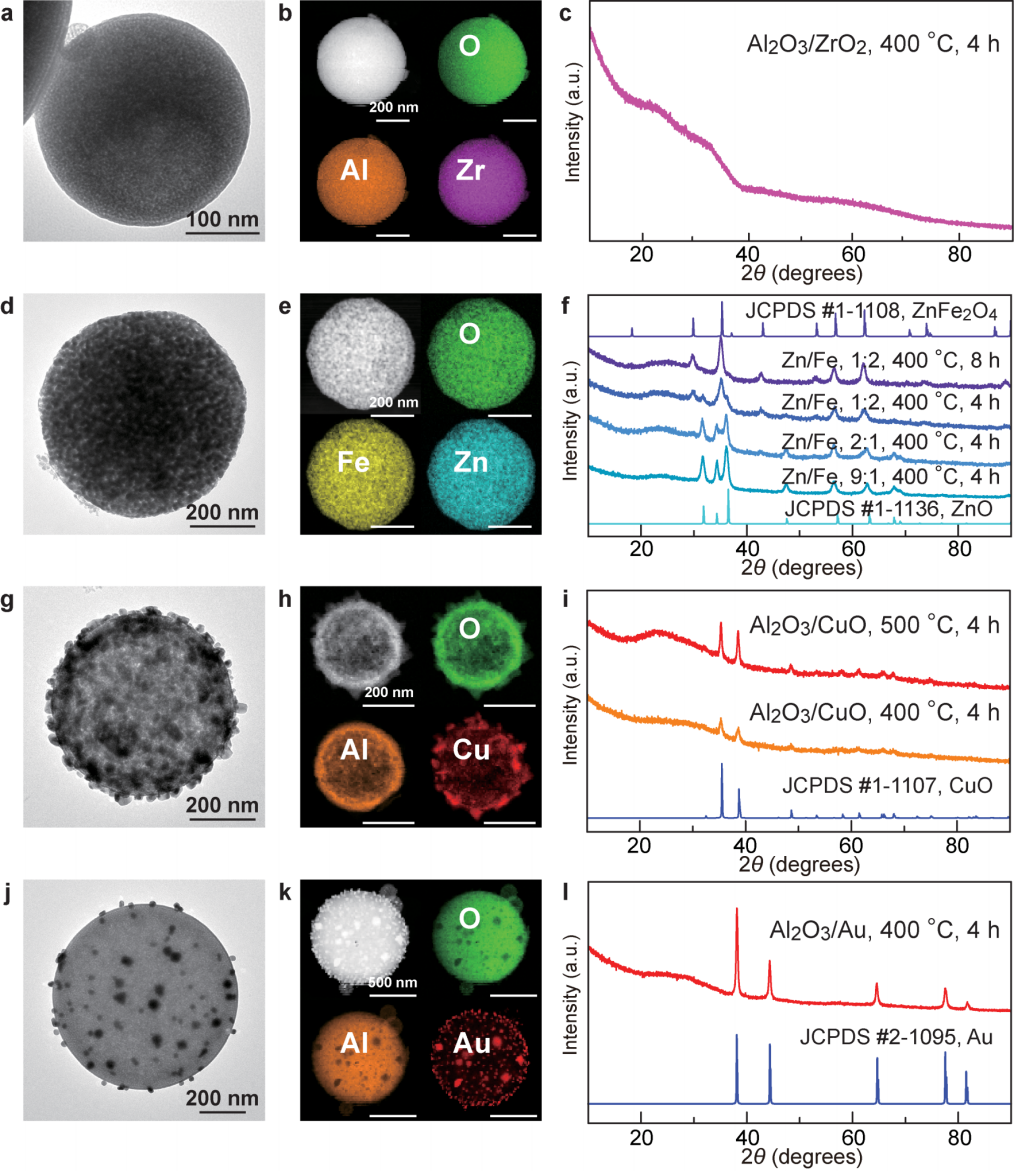
Representative complex mesoporous metal oxide products. (a–c) Mixed Al_2_O_3_–ZrO_2_ microspheres, where both oxides are amorphous. (d–f) Variation from Fe-doped ZnO microspheres to bimetallic ZnFe_2_O_4_ microspheres as the molar ratio between Zn and Fe is changed. (g–h) Mixed Al_2_O_3_–CuO microspheres, where Al_2_O_3_ is amorphous while CuO is crystalline. (j–l) Al_2_O_3_ microspheres loaded with Au nanoparticles. In the first column are the TEM images of the corresponding complex oxide microspheres. In the second column are the HAADF-STEM images (top-left corner) and elemental mappings of the corresponding products. The scale bars in the elemental mappings are the same as that in the HAADF-STEM image for each product. The images in (d) and (e) were acquired on the bimetallic ZnFe_2_O_4_ product with the Zn-to-Fe molar ratio being 1:2. The product was calcined at 400°C for 4 h. In the third columns are the XRD patterns of the products. Some related standard JCPDS patterns are also shown for comparison.

Mesoporous mixed and bimetallic oxides were made by judiciously choosing two different types of metal nitrates and simply adding them together in the precursor solutions. Our approach allowed for the compositions of the precursor solutions to be readily varied to obtain desired mesoporous oxide products. Figure 2a–i show the representative examples of such mesoporous mixed and bimetallic oxide microspheres. The products were carefully characterized by TEM, high-angle annular dark-field scanning transmission electron microscopy (HAADF-STEM), energy-dispersive X-ray (EDX) analysis, elemental mapping and XRD. When Al(NO_3_)_3_⋅9H_2_O and ZrO(NO_3_)_2_⋅4H_2_O were co-added in the precursor solution, the obtained product was amorphous, with Al and Zr uniformly distributed within each microsphere (Fig. 2a–c). This type of mesoporous products is therefore made of two amorphous metal oxides. When Zn(NO_3_)_2_⋅6H_2_O and Fe(NO_3_)_3_⋅9H_2_O were co-used as the precursors, the crystalline phase of the obtained product was found to vary with the Zn-to-Fe molar ratio (Fig. 2d–f). When the Zn-to-Fe molar ratio was 9:1 and 2:1, the crystalline ZnO phase was the dominant component in the products. When the Zn-to-Fe molar ratio was changed to 1:2, the ferrite phase, ZnFe_2_O_4_, became dominant in the product. Its crystallinity could be increased by lengthening the calcination time. Other ferrites, such as MFe_2_O_4_ with M being Co, Cu and Ni, were also successfully made in a similar way, where the dominant crystalline ferrite phases were obtained by increasing the calcination temperature or time (Supplementary Fig. 9). When Al(NO_3_)_3_⋅9H_2_O and Cu(NO_3_)_2_⋅3H_2_O were mixed in the precursor solution, the product was composed of amorphous Al_2_O_3_ and crystalline CuO (Fig. 2g–i). The Al_2_O_3_ ingredient remained amorphous even though the calcination temperature was raised. Then formed CuO nanoparticles were dispersed in the Al_2_O_3_ host.

Besides the preparations of a variety of mesoporous monometallic and complex oxides by choosing proper metal nitrate precursors, nanoscale species of particular functionalities can also be readily introduced into the oxide products by either adding the proper precursors for making nanoscale species or pre-synthesized nanoparticles in the solutions for aerosol spray. The first route was exemplified by adding HAuCl_4_ in the oxide precursor solution. Au nanoparticles were generated during the spray process and further enlarged by calcination. Figure 2j–l show the product prepared in this way, where Au nanoparticles were embedded in mesoporous Al_2_O_3_ microspheres. Besides the amorphous Al_2_O_3_ framework, mesoporous α-Fe_2_O_3_ (Supplementary Fig. 10) and CeO_2_ (Supplementary Fig. 11) microspheres with crystalline walls could also be loaded with Au nanoparticles similarly. The amount of loaded Au nanoparticles could be facilely controlled by adjusting the amount of HAuCl_4_ added in the precursor solution. The second route for the introduction of functionalities was demonstrated by the addition of pre-synthesized nanoscale species. As an example, pre-synthesized ferrite Zn*_x_*Fe_1-*x*_Fe_2_O_4_ nanoparticles were encapsulated in mesoporous Al_2_O_3_ microspheres, which were also co-loaded with Au nanoparticles through the first route (Supplementary Fig. 12). The product was superparamagnetic and could be collected with a small magnet. The superparamagnetic property was inherited from the ferrite nanoparticles.

### Photocatalytic oxygen evolution and gas sensing

**Figure 3:**
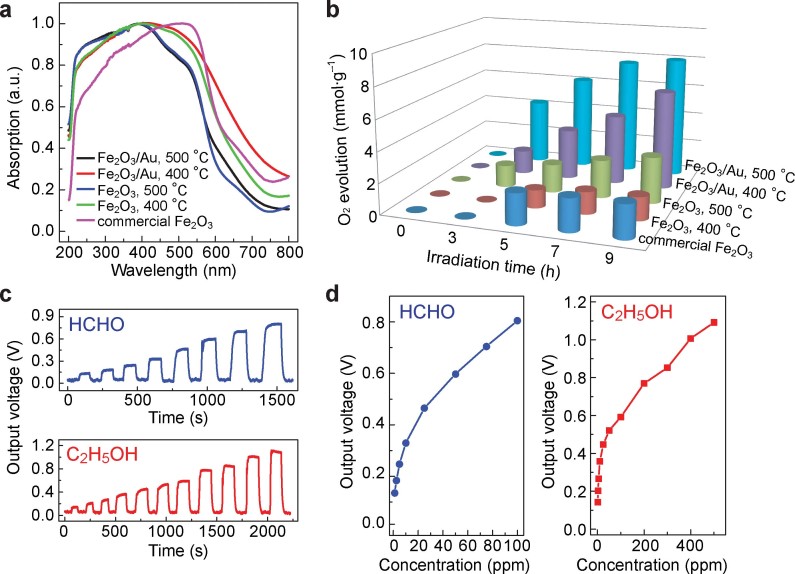
Photocatalytic oxygen evolution and gas sensing performances. The tests were carried out with the different mesoporous Fe_2_O_3_-based products. (a) Normalized absorption spectra of the different Fe_2_O_3_-based products and a commercial Fe_2_O_3_ product. The absorption spectra were determined from UV-visible diffuse reflectance spectroscopy. (b) Oxygen evolution rates as functions of the irradiation time for the different Fe_2_O_3_-based samples. (c) Real-time sensing response curves of the mesoporous α-Fe_2_O_3_ microspheres for formaldehyde (top) and ethanol (bottom), where the concentrations of both types of gas molecules are gradually increased. (d) Output voltage signals as functions of the molecular concentrations for formaldehyde (left) and ethanol (right). The working temperature of the sensing device was 280°C.

To demonstrate the wide potential applications of our aerosol-sprayed mesoporous metal oxides, we studied the photocatalytic oxygen evolution and gas sensing performances of the mesoporous Fe_2_O_3_ products (Fig. 3). α-Fe_2_O_3_, due to its low cost, abundance, and bandgap energy in the visible region (Fig. 3a), has received wide attention for photocatalytic and photoelectrochemical water splitting^27^,^28^. In our photocatalytic measurements, Ag^+^ ions were added to consume photogenerated electrons. When the product was calcined at 500°C, the photocatalytic O_2_ evolution reached 3.0 mmol g^−1^ after 9-h reaction (Fig. 3b). This amount was twice that of the product calcined at 400°C. In comparison, a commercial α-Fe_2_O_3_ product generated 2.2 mmol g^−1^ of O_2_ within 9 h. The differences in the photogenerated amounts of O_2_ can be attributed to the joint effects of the crystallinity and specific surface area. Moreover, when Au nanoparticles were loaded in the mesoporous Fe_2_O_3_ microspheres, the O_2_ evolution amounts were greatly increased. For the products calcined at 400°C and 500°C, 6.5 mmol g^−1^ and 8.0 mmol g^−1^ of O_2_ were generated within 9-h reaction. The resultant largest O_2_ evolution rate, 0.89 mmol h^−1^ g^−1^, is among the highest photocatalytic O_2_ evolution rates that have been reported with various Fe_2_O_3_-based photocatalysts under visible light irradiation (Ref.^29^ and Supplementary Table 2). The enhanced O_2_ evolution rates upon the loading of Au nanoparticles are ascribed to the enhanced charge separation at the interface between the Au nanoparticles and the Fe_2_O_3_ host as well as plasmon-enhanced light absorption by the embedded Au nanoparticles, as have been discussed recently^30^,^31^.

Gas sensing was performed by coating the mesoporous α-Fe_2_O_3_ microspheres on a ceramic tube and measuring the electrical conductance change upon exposure to gas molecules (Supplementary Fig. 13). The sensing signal was measured as a voltage on a sampling resistor. A higher measured voltage corresponded to a larger current, and therefore a smaller resistance, when an oxide was exposed to a particular molecular vapor. The mesoporous Fe_2_O_3_ product was very electrically sensitive to gas molecules owning to its large specific surface area (Fig. 3c&d). The detection limits for formaldehyde and ethanol were as low as 1 ppm by volume, with a signal-to-noise ratio of 3. These detection limits are about one order of magnitude lower than those reported in many other studies^32^,^33^,^34^. The response time, which is defined at the time required for the signal to reach 63% of its stable value, is only ~5 s. Many other types of gas molecules could also be detected with remarkable sensitivities (Supplementary Fig. 14). Meanwhile, the measured voltage signal was found to increase as the working temperature of the sensor was raised (Supplementary Fig. 15). In contrast, the commercial Fe_2_O_3_ product exhibited very poor performance in gas sensing. Almost no response could be recorded on our measurement setup. The high sensitivity of our mesoporous Fe_2_O_3_ product can be explained qualitatively. In general, gas sensing with oxide semiconductors is based on the resistance change upon adsorption of target gas molecules on the surface of the oxide semiconductor. Fe_2_O_3_ is typically an n-type semiconductor. If oxygen molecules are adsorbed on its surface due to exposure to ambient environment, its resistance is increased, because a fraction of electrons are transferred from the oxide to the adsorbed oxygen molecules. When the oxide is exposed to small organic molecules, for example, HCHO, the adsorbed oxygen molecules react with HCHO molecules, electrons are returned back to the oxide, and therefore the resistance is reduced. In our experiments, the Fe_2_O_3_ microspheres are mesoporous. They have a large specific surface area for interaction with gas molecules. Moreover, the pores allow for gas molecules to diffuse into the microspheres. As a result, the mesoporous Fe_2_O_3_ microspheres offer a high sensitivity for gas sensing.

## Conclusion

In summary, our experiments show that the use of low-cost, versatile metal nitrate salts as precursors in conjunction with aerosol spray-induced, block copolymer-directed assembly offers a facile yet powerful means for the preparation of a variety of mesoporous monometallic, multimetallic, and complex metal oxides. Our method allows for easy doping and formation of oxide mixtures, multimetallic oxides, as well as introduction of additional functional nanoscale species. Such modifications are often resorted to for changing or improving the optical and electronic properties of metal oxides, but they are often difficult to be realized with conventional wet-chemistry or solid-state preparation methods. Our method is expected to bring many advances and improvements in the performances of enormous materials and devices based on metal oxides.

## Methods

### Mesoporous metal oxide preparation

To prepare the precursor solution, (ethylene oxide)_20_-(propylene oxide)_70_-(ethylene oxide)_20_ (Pluronic P123) or (ethylene oxide)_106_-(propylene oxide)_70_-(ethylene oxide)_106_ (Pluronic F127) triblock copolymer was first dissolved in absolute ethanol by stirring. After the copolymer was completely dissolved, a metal nitrate salt was added in the solution, followed by vigorous stirring for 10 min. The resultant transparent solution was then transferred to a household ultrasonic humidifier for aerosol generation. The liquid droplets were carried by nitrogen into a tube furnace that was set at a particular temperature. The product was collected through filtration. To prepare complex oxides or oxide-based hybrids, multiple metal nitrates at controlled molar ratios, dissolvable molecular precursors for non-oxides, and/or dispersible pre-synthesized nanoscale species were added together in the ethanolic solution of the copolymer. The collected products were calcined in air to remove the copolymer surfactant. More preparation details are provided in Supplementary methods.

### Characterization

The sprayed products were examined by powder XRD (SmartLab Diffractometer, 40 kV, 40 mA), SEM (FEI QF400, attached with an EDX analysis system), TEM (FEI TS12, 120 kV), high-resolution TEM (FEI TF20, 200 kV), UV-visible diffuse reflectance spectroscopy (UV-2450, Shimadzu), and HAADF-STEM imaging and elemental mapping (FEI TF20, 200 kV). Nitrogen sorption isotherms were measured on a Micromeritics TriStar 3000 system at the liquid-nitrogen temperature. The specific surface area and pore size distribution were analyzed according to the Brunauer–Emmett–Teller and Barrett–Joyner–Halenda model, respectively.

### Photocatalytic and gas sensing measurements

Photocatalytic water oxidation was performed in an aqueous AgNO_3_ solution in a photocatalytic water-splitting system, where AgNO_3_ functioned as a sacrificial agent. The reaction was under visible light illumination (*λ* > 420 nm). The sensing device was made of a ceramic tube with two printed gold electrodes. The mesoporous oxide powders were coated on the tube at a thickness of several ten micrometers. The working temperature of the sensing tube was controlled with a resistance heater. The sensing measurements were carried out in a home-designed chamber with an electrical measurement system. The gas vapor was injected into the chamber with a syringe and mixed automatically with air. The concentration of the gas vapor was estimated by taking into account the saturated vapor pressure of the gas at a given temperature, the injected vapor volume, and the chamber volume. More details for the photocatalytic and sensing measurements are provided in Supplementary methods.

## Author Contributions

J.F.W., T.M. and B.Y.G. conceived the project. L.K., T.M. and J.X.W. performed the experiments. C.H.F. contributed to the TEM characterizations. Z.H.S. performed the nitrogen sorption measurements. L.K., J.X.W. and J.F.W. wrote the paper.

## Supplementary Material

Supplementary Information Supplementary information

## Figures and Tables

**Table 1:** Preparation and pore structure parameters of the mesoporous monometallic oxides

Oxide	Precursor	Thermal decomposition temperature (°C)	Furnace temperature (°C)	Surface area (m^2^ g^−1^)	Pore volume (cm^3^ g^−1^)
Al_2_O_3_	Al(NO_3_)_3_⋅9H_2_O	200	400	118	0.18
Cr_2_O_3_	Cr(NO_3_)_3_⋅*x*H_2_O	100	400	101	0.12
Mn_3_O_4_	Mn(NO_3_)_2_⋅4H_2_O	160–200	400	87	0.33
Fe_2_O_3_	Fe(NO_3_)_3_⋅9H_2_O	125	400	194	0.52
Co_3_O_4_	Co(NO_3_)_2_⋅6H_2_O	200–240	500	56	0.21
ZnO	Zn(NO_3_)_2_⋅6H_2_O	350–380	500	38	0.18
Ga_2_O_3_	Ga(NO_3_)_3_⋅4H_2_O	200	400	135	0.25
ZrO_2_	ZrO(NO_3_)_2_⋅4H_2_O	200	400	116	0.16
CeO_2_	Ce(NO_3_)_3_⋅9H_2_O	200	400	147	0.19
MgO	Mg(NO_3_)_2_⋅6H_2_O	300–330	500	39	0.19
NiO	Ni(NO_3_)_2_⋅6H_2_O	270–310	500	50	0.17
CuO	Cu(NO_3_)_2_⋅3H_2_O	155–160	400	19	0.10
WO_3_	H_2_WO_4_		400	18	0.04
