# Supplementary material for: Aerosol-spray diverse mesoporous metal oxides from metal nitrates
Source: Sci Rep. 2015 Apr 21;5:9923. doi: 10.1038/srep09923 (PMC4404711; doi:10.1038/srep09923)
Supplement: Supplementary Information [file srep09923-s1.pdf]

Supplementary information

Aerosol-spray diverse mesoporous metal oxides from metal nitrates

Long Kuai[1,2], Junxin Wang[2], Tian Ming[2], Caihong Fang[2], Zhenhua Sun[3], Baoyou Geng[1] & Jianfang Wang[2]

[1]College of Chemistry and Materials Science, The Key Laboratory of Functional Molecular Solids, Ministry of Education, Anhui Laboratory of Molecular-Based Materials, Center for Nano Science and Technology, Anhui Normal University, Wuhu 241000, China, [2]Department of Physics, The Chinese University of Hong Kong, Shatin, Hong Kong SAR, China, [3]Shenyang National Laboratory for Materials Science, Institute of Metal Research, Chinese Academy of Sciences, Shenyang 110016, China.

Supplementary figures

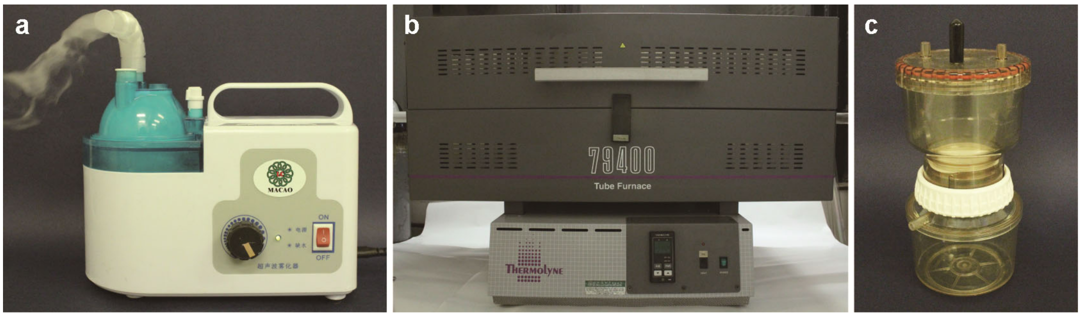

Supplementary Figure 1 | Apparatuses for ultrasonic aerosol spray. (a) Household ultrasonic humidifier. (b) Tube furnace. (c) Filter for sample collection.

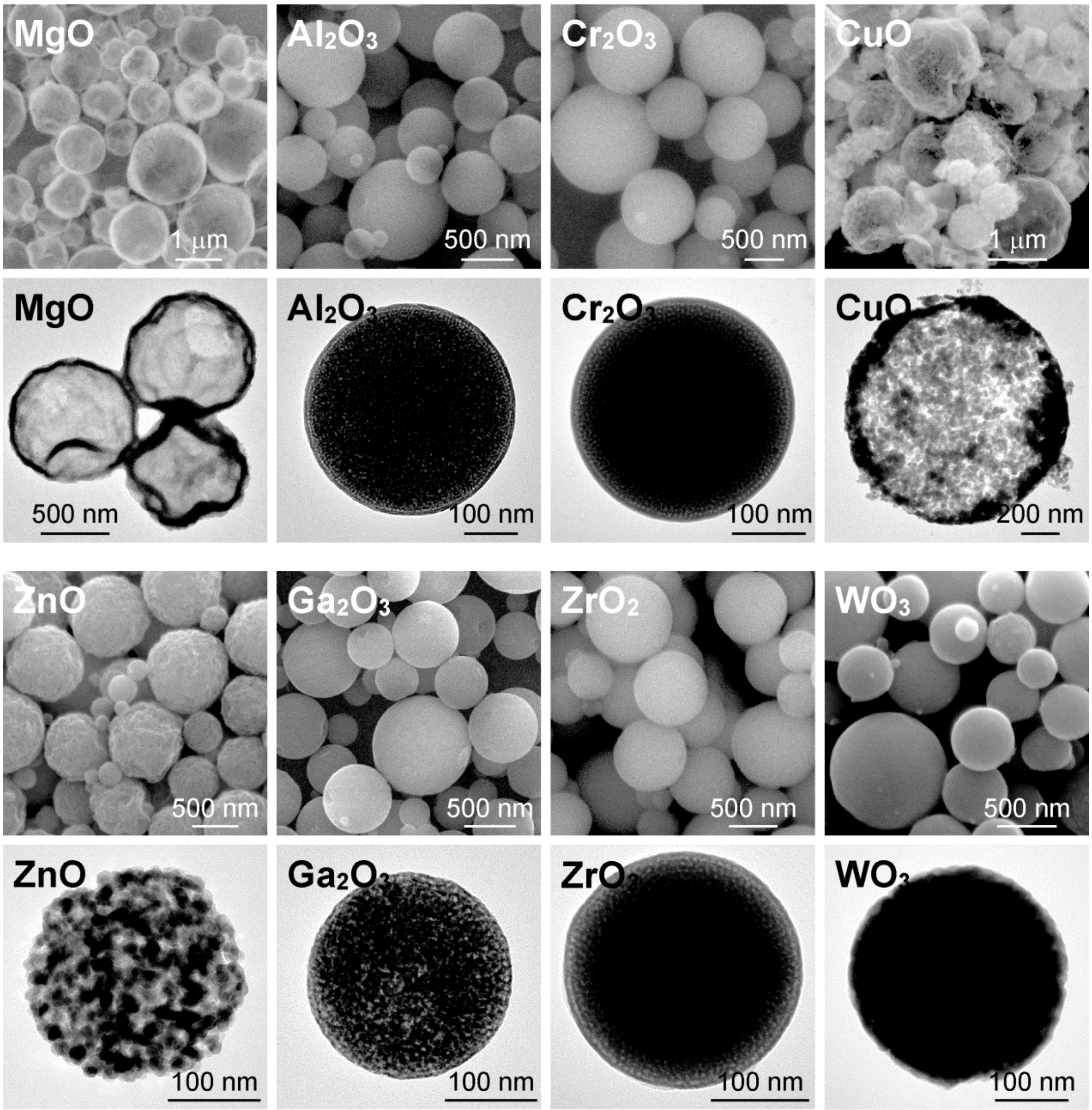

Supplementary Figure 2 | Mesoporous monometallic oxide products. In the first and third rows are the SEM images. In the second and fourth rows are the TEM images. The shown products include MgO, Al_2O_3, Cr_2O_3, CuO, ZnO, Ga_2O_3, ZrO_2, and WO_3. They were all calcined at 400 °C for 4 h.

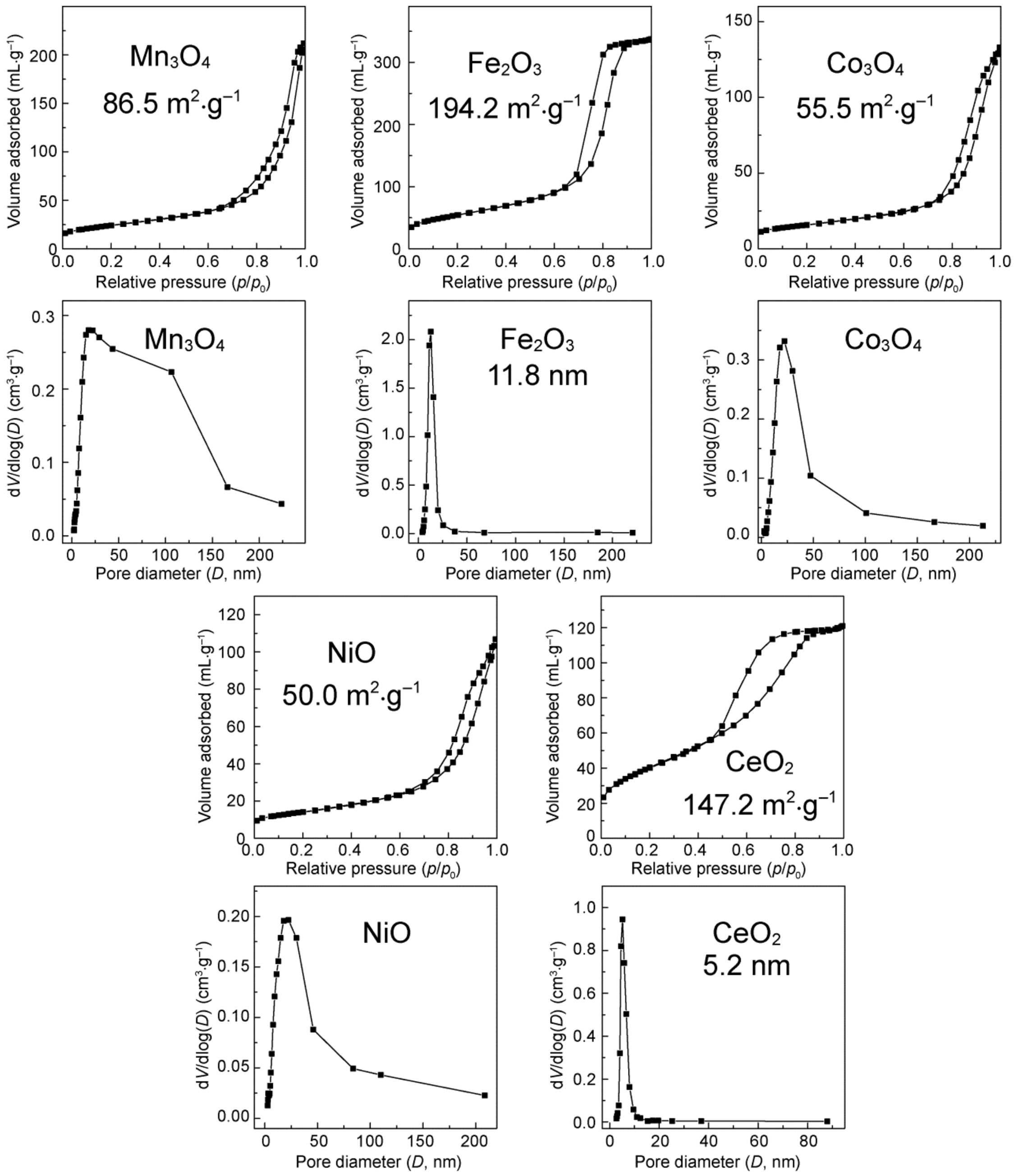

Supplementary Figure 3 | Porous characteristics of the monometallic products. In the first and third rows are the nitrogen sorption isotherms. In each isotherm, the lower branch is for adsorption, and the upper branch is for desorption. In the second and fourth rows are the corresponding Barrett−Joyner−Halenda pore size distributions. The shown data are for the products of Mn_3O_4, Fe_2O_3, Co_3O_4, NiO, and CeO_2, which were calcined at 400 °C for 4 h.

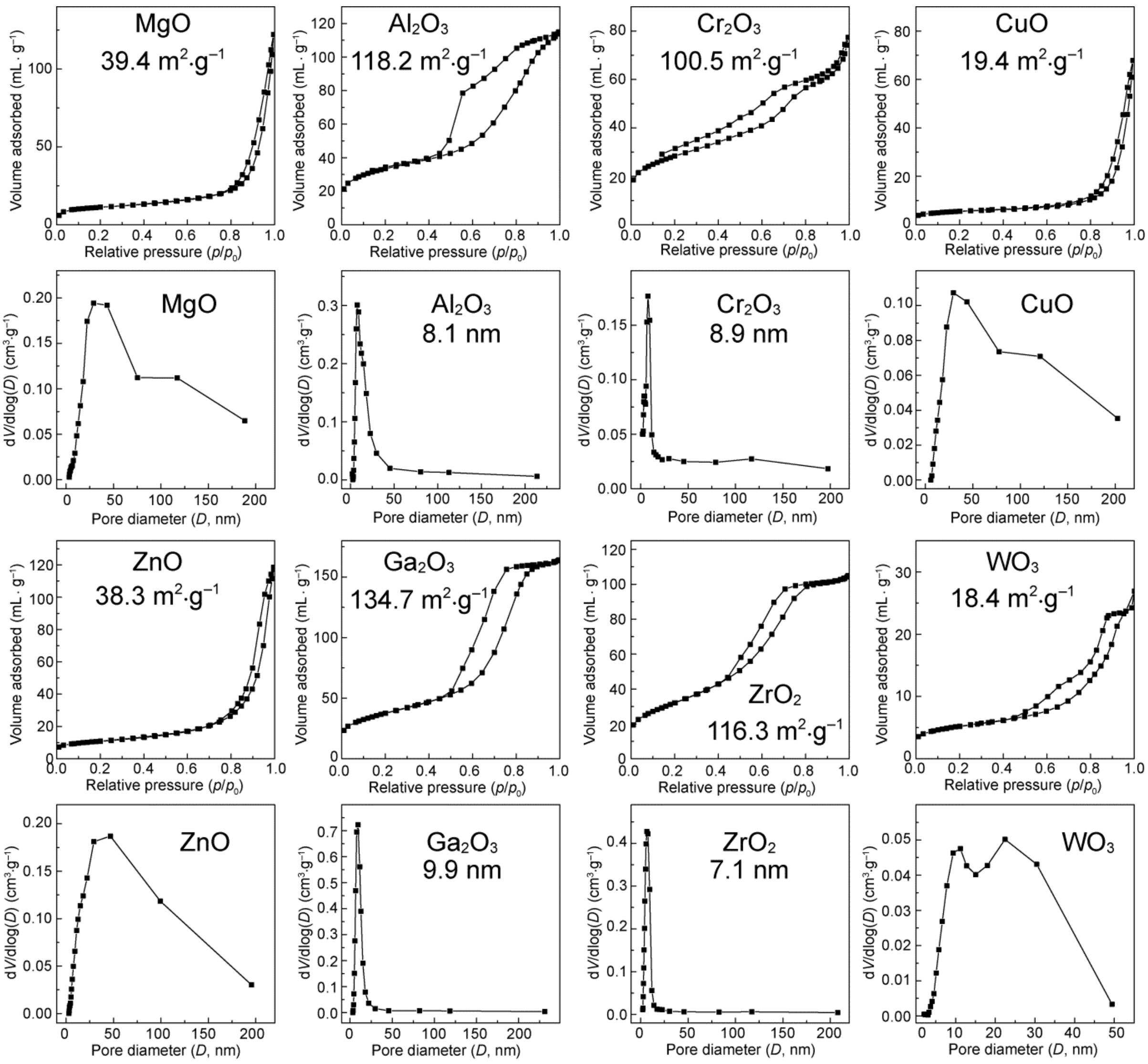

Supplementary Figure 4 | Porous characteristics of the monometallic products. In the first and third rows are the nitrogen sorption isotherms. In each isotherm, the lower branch is for adsorption, and the upper branch is for desorption. In the second and fourth rows are the corresponding Barrett−Joyner−Halenda pore size distributions. The shown data are for the products of MgO, Al_2O_3, Cr_2O_3, CuO, ZnO, Ga_2O_3, ZrO_2, and WO_3, which were calcined at 400 °C for 4 h.

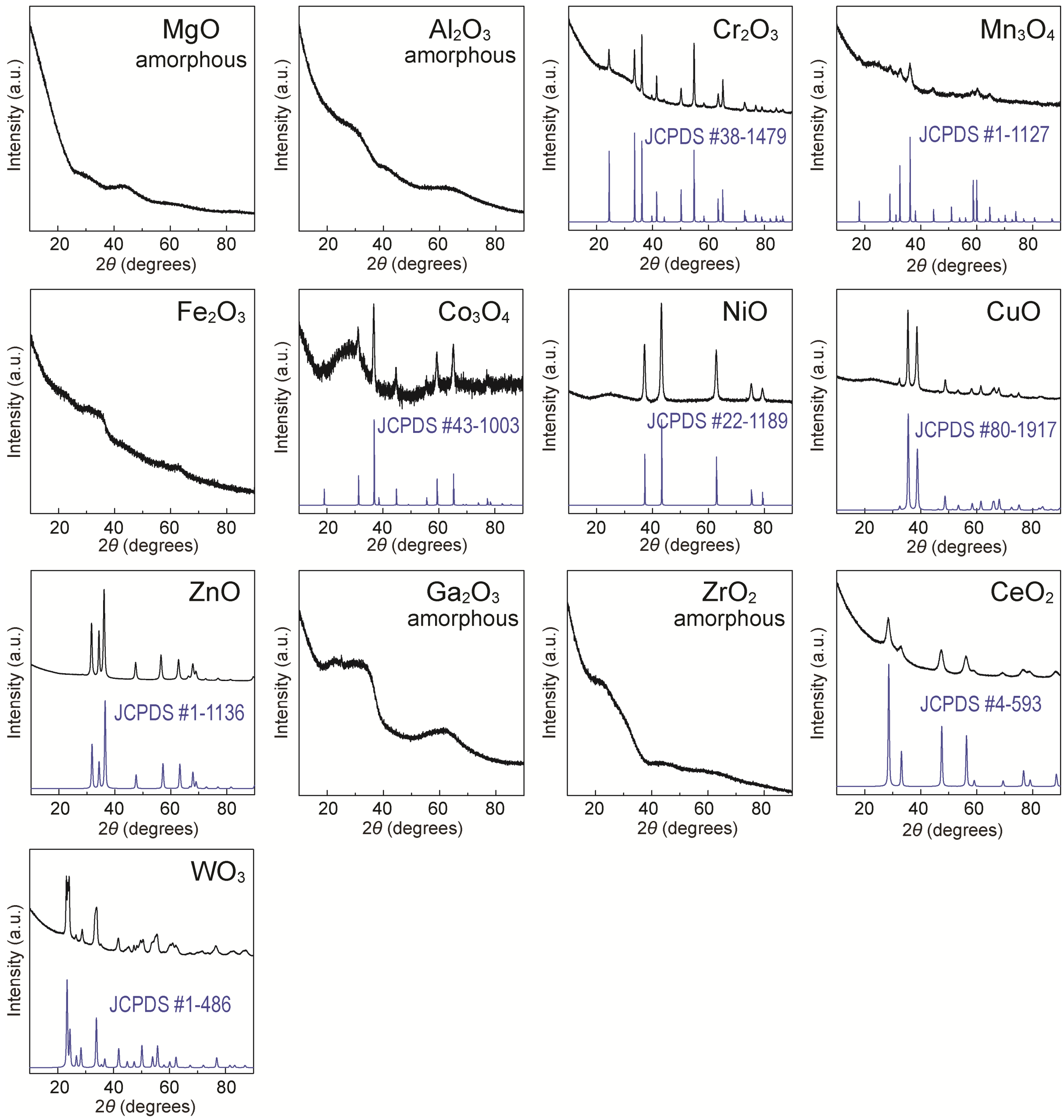

Supplementary Figure 5 | XRD patterns of the mesoporous monometallic products. The XRD patterns of all of the 13 types of the monometallic products are shown. All of the samples were calcined at 400 °C for 4 h. The mesoporous MgO, Al_2O_3, Fe_2O_3, Ga_2O_3, and ZrO_2 products are amorphous, while the mesoporous Cr_2O_3, Mn_3O_4, Co_3O_4, NiO, CuO, ZnO, CeO_2, and WO_3 products are polycrystalline. The most matching JCPDS pattern is also shown for each polycrystalline sample.

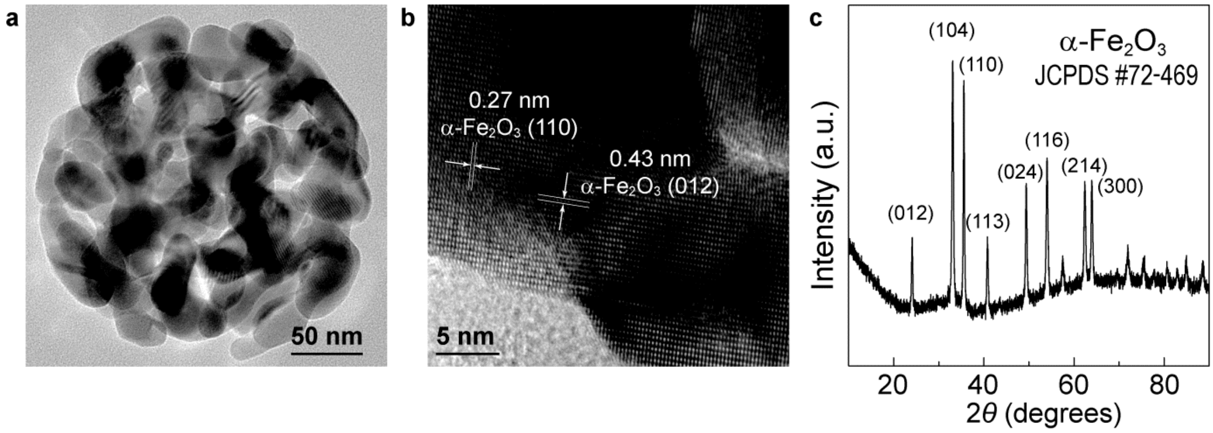

Supplementary Figure 6 | Structural characterization of the α-Fe_2O_3 product. (a) TEM image of a single microsphere. (b) HRTEM image recorded in the edge region of a single microsphere. (c) XRD pattern of the product. The diffraction peaks are indexed according to JCPDS #72-469.

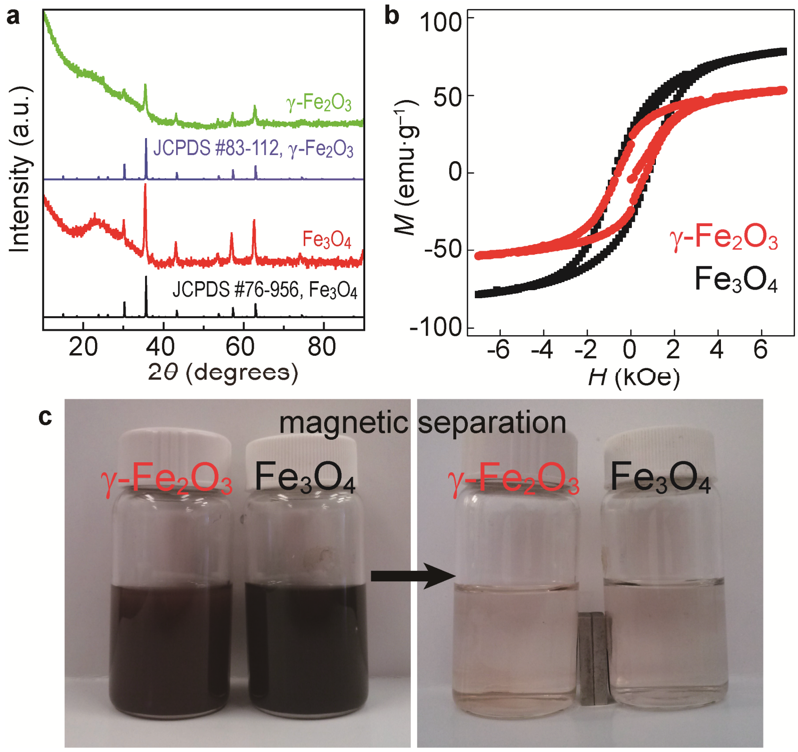

Supplementary Figure 7 | Fe_3O_4 and γ-Fe_2O_3 products. (a) XRD patterns. The most matching JCPDS patterns are also shown for comparison. (b) Room-temperature magnetic

hysteresis curves. (c) Digital photographs illustrating the magnetic adsorption of the particles in the solutions to the side walls of the glass containers with a magnet.

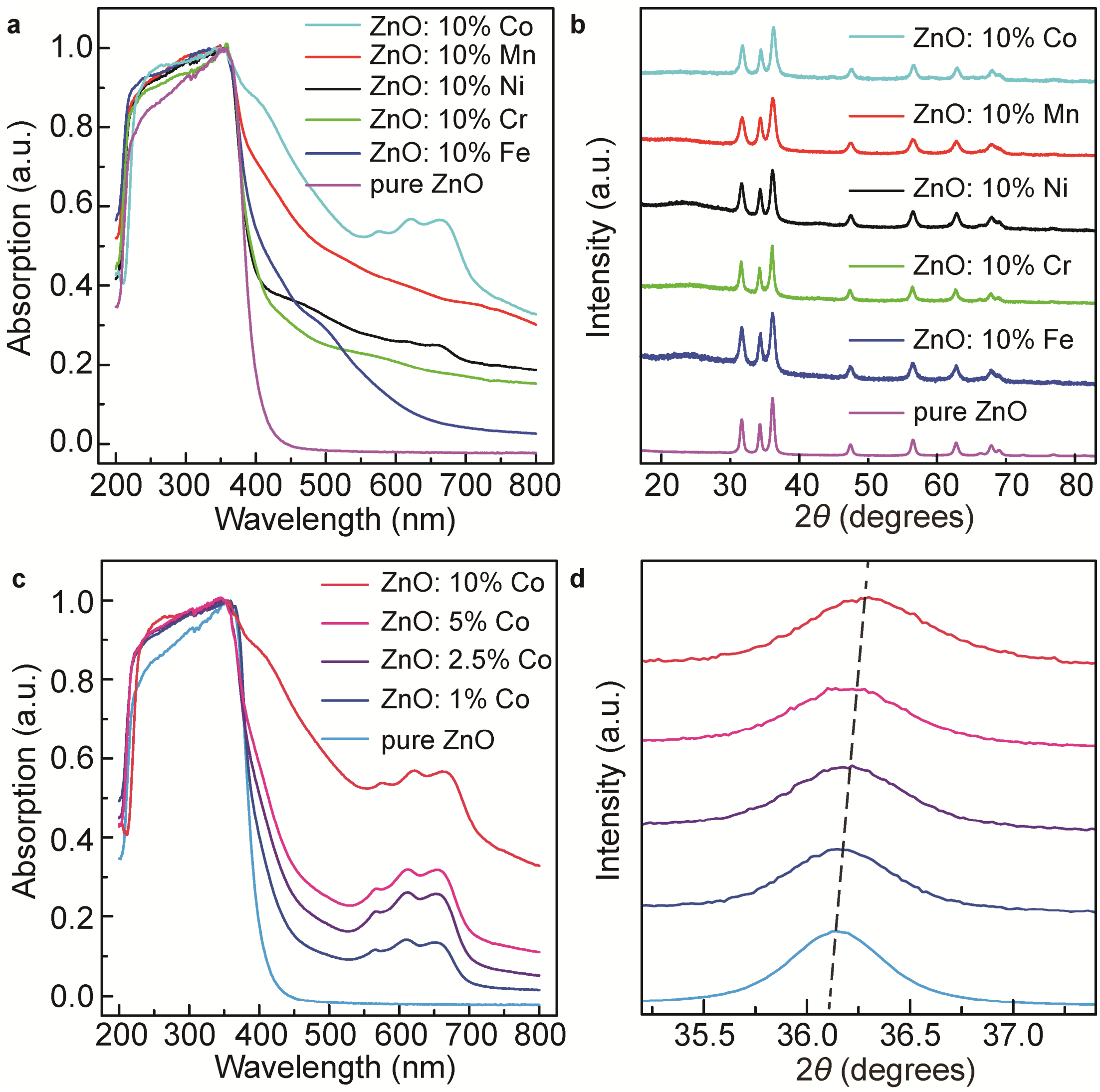

Supplementary Figure 8 | Doped ZnO products. (a) Normalized absorption spectra of the pure ZnO product and the ZnO products doped with Co, Mn, Ni, Cr and Fe at 10 mol%, respectively. The absorption spectra of the solid products were determined by UV-visible diffuse reflectance spectroscopy. (b) XRD patterns of the same products as shwon in (a). (c) Normalized absorption spectra of the pure ZnO product and the ZnO products doped with Co at concentrations of 1.0 mol%, 2.5 mol%, 5.0 mol% and 10.0 mol%, respectively. (d) Enlarged XRD patterns of the same products as shown in (c) in the 2θ range around 36°.

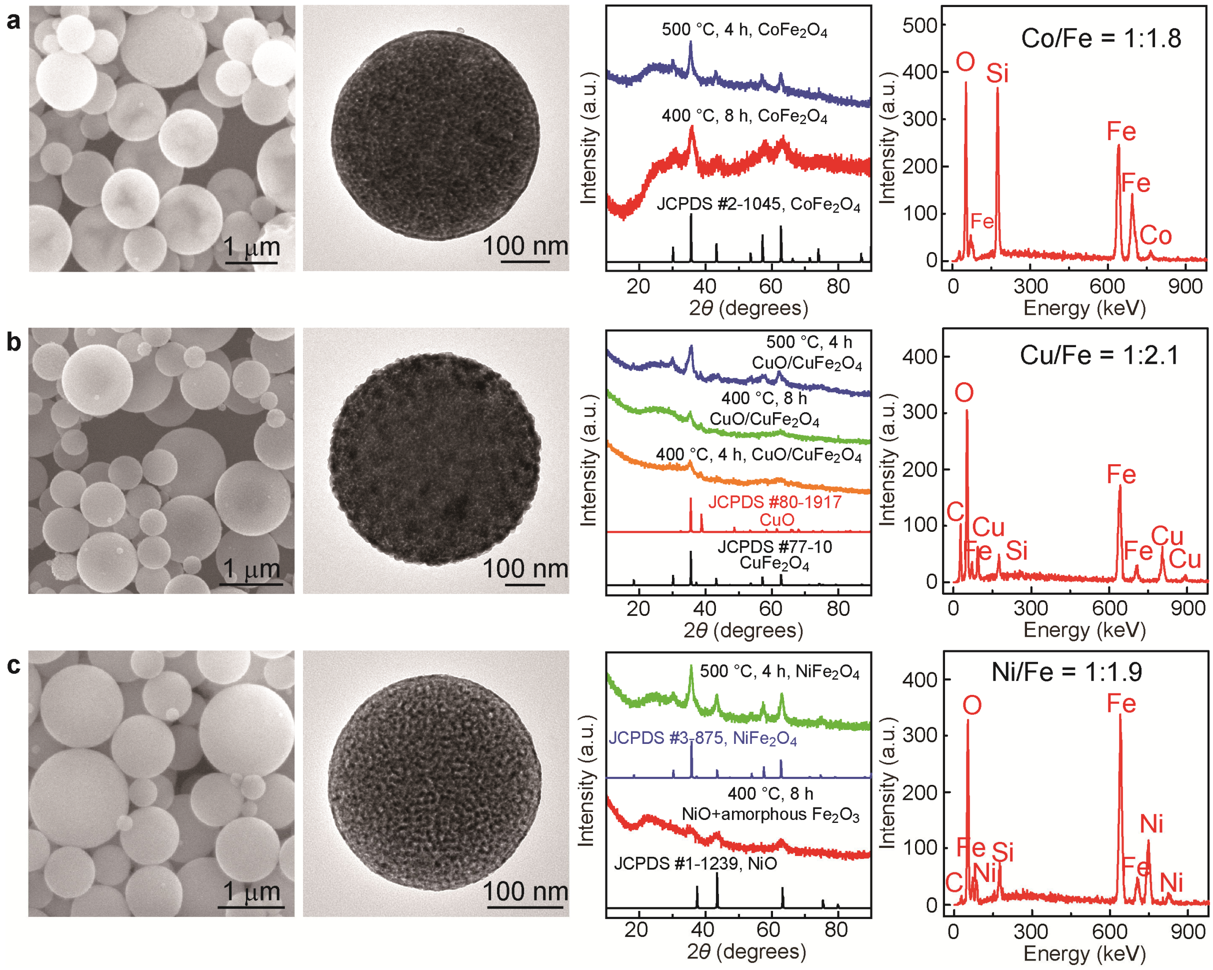

Supplementary Figure 9 | Mesoporous bimetallic oxides. (a) Co–Fe oxides. (b) Cu–Fe oxides. (c) Ni–Fe oxides. In the leftmost column are the SEM images. In the second column are the TEM images of the single mesoporous bimetallic microspheres. In the third column are the XRD patterns of the products obtained by calcination under different conditions. The most matching JCPDS patterns are also shown. In the fourth column are the EDX spectra of the mesoporous bimetallic oxides. The molar ratios between the respective metals to Fe determined from the EDX analyses are also given. The SEM and TEM images were taken on the products that were calcined at 400 °C for 8 h.

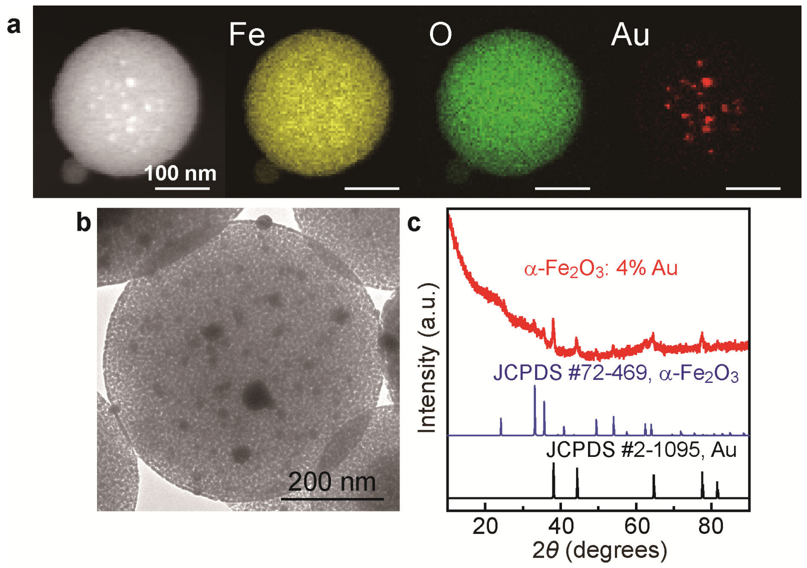

Supplementary Figure 10 | Au nanoparticle-loaded mesoporous α-Fe_2O_3 microspheres. (a) HAADF-STEM image (leftmost) and elemental mappings of a single microsphere. The scale bars are the same as that shown on the HAADF-STEM image. (b) TEM image of the product. (c) XRD pattern of the product. The JCPDS patterns of Au and α-Fe_2O_3 are also shown for comparison.

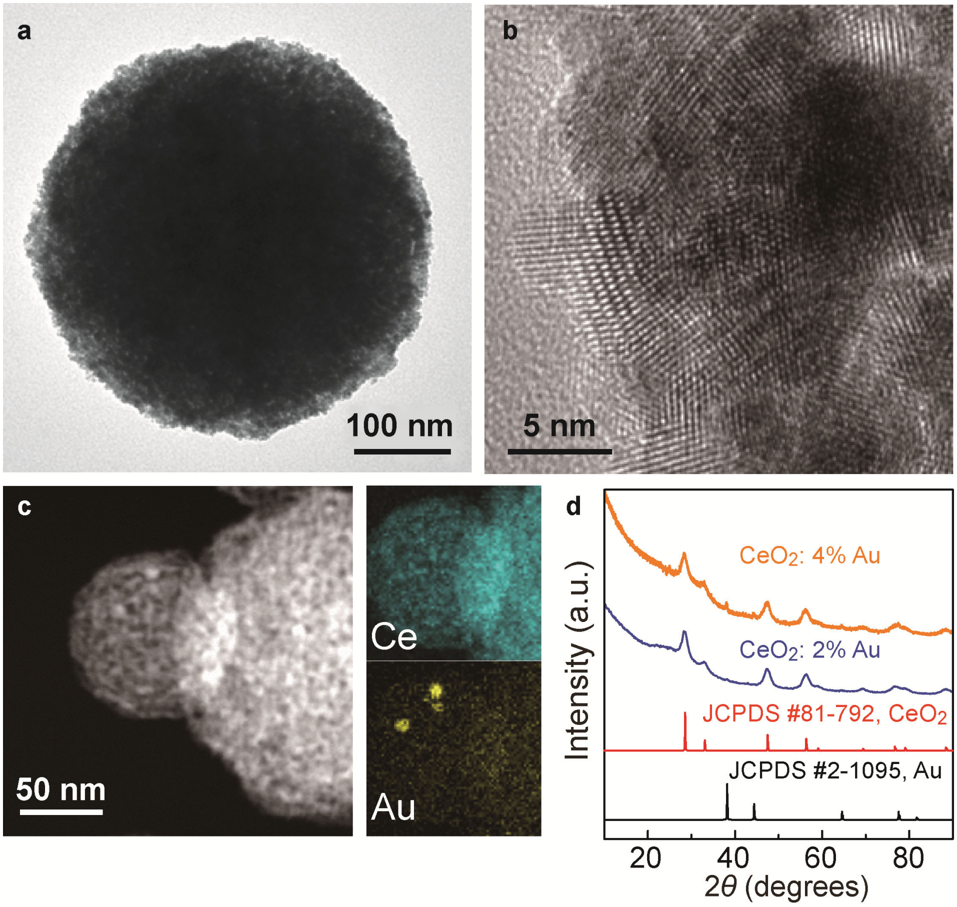

Supplementary Figure 11 | Au nanoparticle-loaded mesoporous CeO_2 microspheres. (a) TEM image of a single microsphere. (b) HRTEM image taken from the edge region of a single microsphere. (c) HAADF-STEM image (left side) and elemental mappings of the product. The elemental mapping images have the same magnification as that of the HAADF-STEM image. (d) XRD patterns of the mesoporous CeO_2 microsphere samples loaded with Au nanoparticles at two different concentrations. The standard JCPDS patterns of Au and CeO_2 are also shown for comparison.

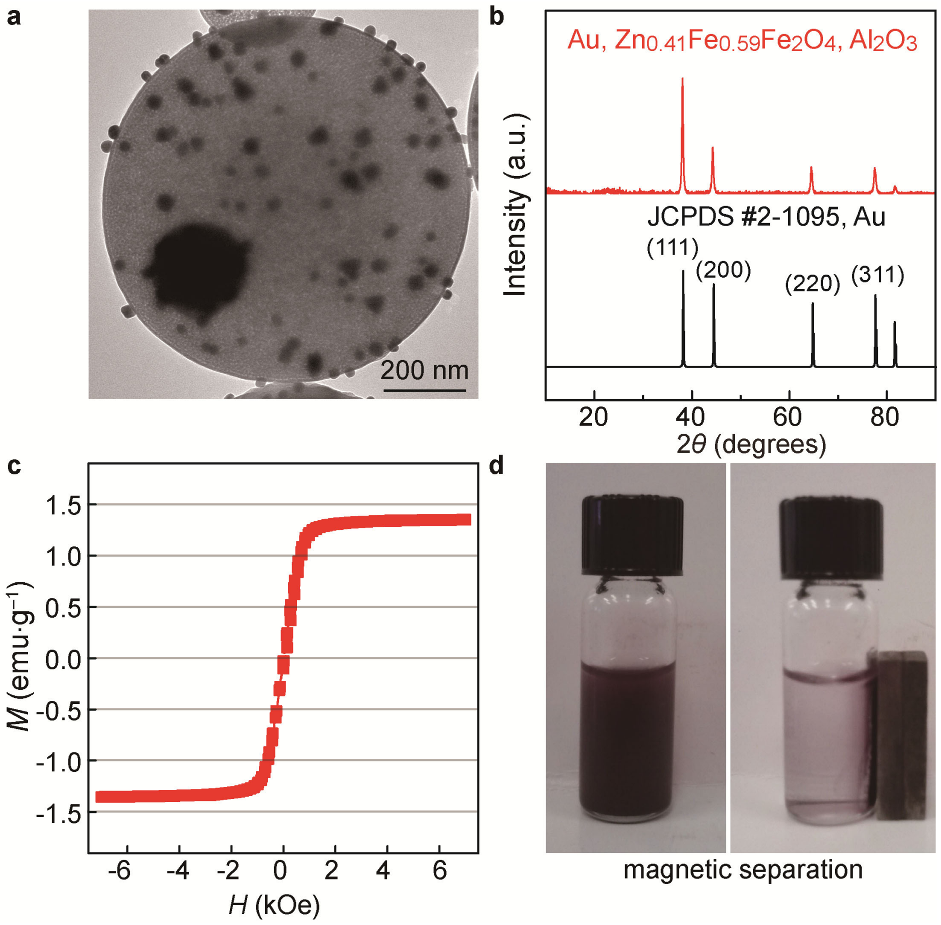

Supplementary Figure 12 | Loaded mesoporous Al_2O_3 microspheres. The microspheres are co-loaded with magnetic $Zn_{0.41}Fe_{0.59}Fe_2O_4$ nanoparticles and plasmonic Au nanoparticles. (a) TEM image of a single microsphere. (b) XRD pattern of the product. The standard JCPDS pattern of gold is also shown for comparison. The observation that the diffraction peaks of the magnetic $Zn_{0.41}Fe_{0.59}Fe_2O_4$ nanoparticles are not present on the XRD pattern of the product is probably due to the small amount of the magnetic nanoparticles in the microspheres. (c) Room-temperature magnetic hysteresis curve of the product. (d) Digital photographs of the product dispersed in aqueous solutions in the absence and presence of a magnet, respectively.

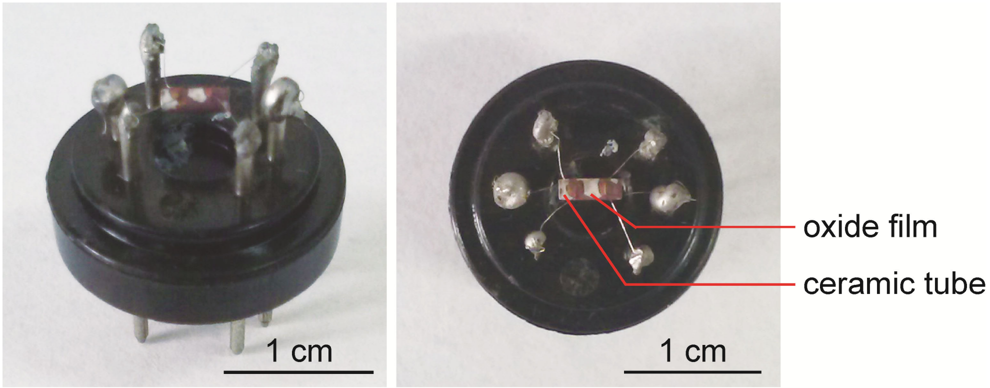

Supplementary Figure 13 | Digital photographs of the gas sensing device. (a) Side view. (b) Top view. There are three pairs of electrodes. They were used for sensing signal measurements, resistive heating of the sensing device, and working temperature measurements, respectively.

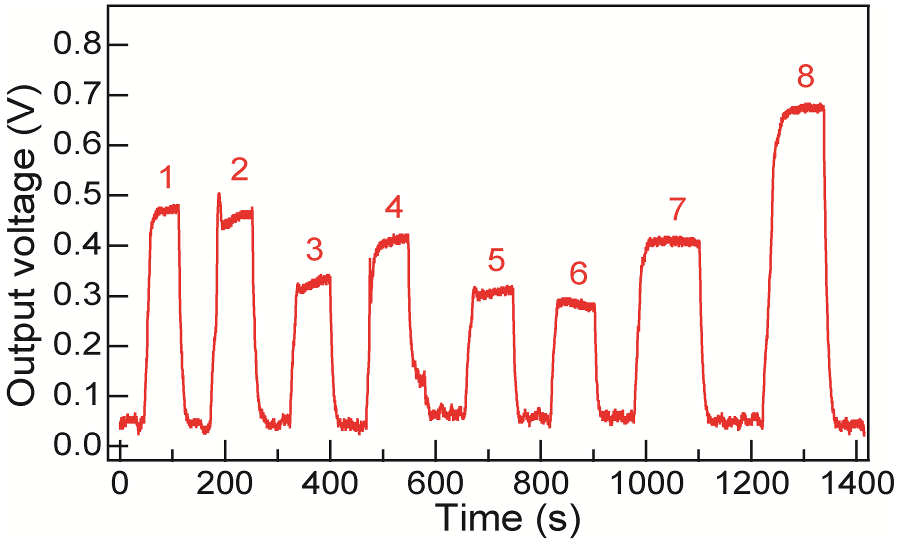

Supplementary Figure 14 | Real-time sensing response curve. The curve was acquired with the mesoporous α-Fe_2O_3 microsphere sample, which was exposed to various types of gas molecules. The sensor was exposed to 100 ppm of (1) ethanol, (2) acetone, (3) methanol, (4) toluene, (5) benzene, (6) ammonia, (7) isopropanol, and (8) formaldehyde, respectively. The working temperature of the sensor was maintained at 280 °C. After the detection of one type of gas molecules, the measurement chamber was pumped to remove the gas molecules.

Dry air was then supplied, followed with the injection of a different type of gas molecules for detection.

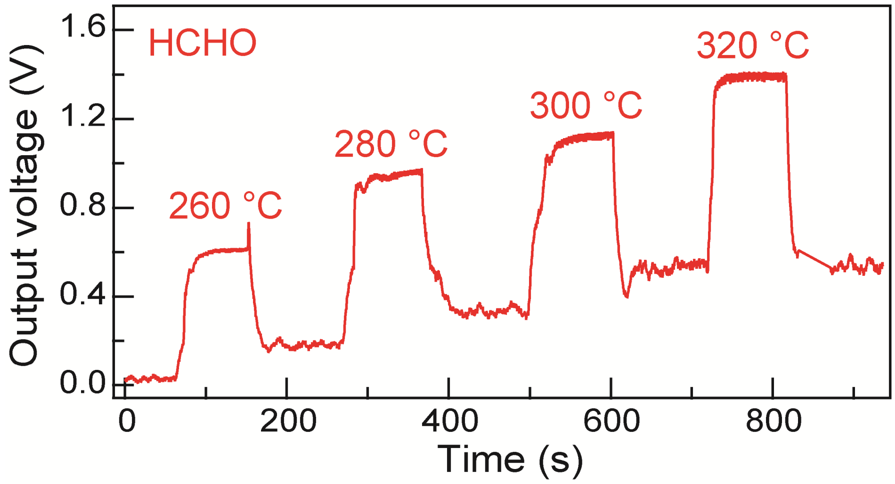

Supplementary Figure 15 | Temperature effect on the gas sensing. The real-time sensing response curve was measured when the mesoporous α-Fe_2O_3 microsphere sample was exposed to formaldehyde at different temperatures. The concentration of formaldehyde was kept at 100 ppm. After the detection at one temperature, the measurement chamber was pumped, followed with the supply of dry air. The working temperature of the sensor was then raised to a higher temperature, followed with the injection of formaldehyde for another measurement.

Supplementary tables

Supplementary Table 1: References for the thermal decomposition temperatures of the nitrate precursors

Precursor	Reference
$Al(NO_3)_3{\cdot}9H_2O$	1–5
$Cr(NO_3)_3{\cdot}xH_2O$	6–9
$Mn(NO_3)_2{\cdot}4H_2O$	4,6,10,11
$Fe(NO_3)_3{\cdot}9H_2O$	4,12–14
$Co(NO_3)_2{\cdot}6H_2O$	2,4,6,15–17
$Zn(NO_3)_2{\cdot}6H_2O$	2,4,6,18
$Ga(NO_3)_3{\cdot}4H_2O$	4,6,19,20
$ZrO(NO_3)_2{\cdot}4H_2O$	4
$Ce(NO_3)_3{\cdot}9H_2O$	4,21,22
$Mg(NO_3)_2{\cdot}6H_2O$	4,6,15,23
$Ni(NO_3)_2{\cdot}6H_2O$	4,6,13
$Cu(NO_3)_2{\cdot}3H_2O$	4,6,18,24
H_2WO_4	

Supplementary Table 2: Representative oxygen evolution rates obtained with Fe_2O_3-based photocatalysts

Sample	Morphology and size	Light source	Oxygen evolution rate (mmol h^{-1} g^{-1})	Reference
4 wt% Au/α-Fe_2O_3, 20 mg	Mesoporous microspheres, 0.2–2 μm	300 W Xe lamp, visible light (λ > 420 nm)	0.89	This work
α-Fe_2O_3/rGO, 6 mg	Hybrid sheets with Fe_2O_3 nanoparticles of 41 nm in size	300 W Xe lamp, AM 1.5G filter	0.75	25
α-Fe_2O_3, 5.6 mg	Nanoparticles of 5 nm in size	300 W Xe lamp, visible light (λ > 400 nm)	0.72	26
Sonic α-Fe_2O_3, 5.6 mg	Nanoparticles of 5–20 nm in size	300 W Xe lamp, visible light (λ > 400 nm)	0.7	26
Bulk α-Fe_2O_3, 5.6 mg	Nanoparticles of 50–300 nm in size	300 W Xe lamp, visible light (λ > 400 nm)	0.23	26
α-Fe_2O_3/Mn_3O_4/graphene, 5 mg	Hybrids with Fe_2O_3 nanorods of 100 nm in diameter and 400 nm in length	150 W Xe lamp, full light	0.141	27
α-Fe_2O_3, 10 mg	Hollow spheres (800 nm in diameter) with ultrathin sheets (1.5 nm in thickness)	300 W Xe lamp, visible light (λ >420 nm)	0.07	28
α-Fe_2O_3/clay, 20 mg	Fe_2O_3 (30–60 nm) embedded in clay	150 W halogen lamp	0.022	29

Supplementary methods

Materials. All chemicals were purchased from Sigma-Aldrich, Aladdin, Fisher Scientific, or Scharlau, and used as received.

Synthesis of mesoporous monometallic oxides. A metal nitrate salt (2 mmol) together with F127 or P123 (0.25 g) was completely dissolved in absolute ethanol (30 mL). The obtained solution was transferred into a household ultrasonic humidifier (1.7 MHz, 30 W) for aerosol

spray. The generated mist was carried by N_2 into a glass or quartz tube that was placed in a 90-cm-long tube furnace. The sizes of the liquid droplets generated under our experimental conditions were estimated to be 3 −8 μm. The temperature of the furnace was pre-set at 400 °C or 500 °C. The product was collected with a filter that was connected to a mechanical vacuum pump or a water aspirator. P123 was used for the synthesis of mesoporous CeO_2, Mn_3O_4, Co_3O_4, and CuO, while F127 was used for the synthesis of the other nine types of mesoporous monometallic oxides. In addition, for the preparation of mesoporous WO_3, H_2WO_4 was used as the precursor. Because H_2WO_4 is difficult to be dissolved in ethanol, H_2WO_4 (2 mmol) was first dissolved in H_2O_2 (6 mL, 30 wt%) under ultrasonication to give a light yellow gel solution, which was thereafter added in ethanol (30 mL) together with F127 (0.25 g).

Synthesis of doped mesoporous ZnO microspheres. $Zn(NO_3)_2$, $M(NO_3)_x$ (M = Co, Ni, Mn, Fe or Cr, 10 mol%), and F127 (0.25 g) were co-dissolved in absolute ethanol (30 mL). The total molar amount of the metals was 2 mmol. The doping molar percentage ($n_M/(n_M + n_{Zn})*100\%$) was accounted relative to the total molar amount of the metals. Ni, Mn, Fe and Cr were doped at 10 mol%, while Co doping was varied from 1 mol% to 10 mol%. After complete dissolution, the precursor solution was subjected to the same aerosol spray and calcination processes as described above for mesoporous monometallic oxides.

Synthesis of mesoporous mixed and bimetallic oxides. The synthesis was similar to that of the mesoporous monometallic oxides except that two types of metal nitrate salts were used. For example, $M(NO_3)_2$ (M = Zn, Cu, Ni and Co) and $Fe(NO_3)_3$ were utilized to prepare mesoporous $M_xFe_yO_4$ (M = Zn, Cu, Ni and Co) microspheres; $Al(NO_3)_3$ and $ZrO(NO_3)_2$ were

employed to synthesize mesoporous Al–Zr–O microspheres; $Al(NO_3)_3$ and $Cu(NO_3)_2$ were used to prepare mesoporous CuO/Al_2O_3 microspheres.

Synthesis of Au-loaded mesoporous Al_2O_3 and Fe_2O_3 microspheres. The synthesis procedure was similar to that of the mesoporous monometallic oxides except that an ethanolic $HAuCl_4$ solution (2.86 g) was additionally supplied in the precursor solution. The $HAuCl_4$ solution was pre-made by dissolving $HAuCl_4$ (1 g) in absolute ethanol (80 g). The loading amount of Au nanoparticles was adjusted to be ~4 mol% ($n_{Au}/(n_{Au} + n_M)*100\%$) relative to the total molar amount of the metals for both mesoporous Al_2O_3 and Fe_2O_3 microspheres.

Synthesis of loaded mesoporous Al_2O_3 microspheres. Pre-synthesized magnetic $Zn_{0.41}Fe_{0.59}Fe_2O_4$ nanoparticles were chosen to be loaded in mesoporous Al_2O_3 microspheres. Their synthesis followed a reported method[30]. Typically, $FeCl_3{\cdot}6H_2O$ (0.421 g), $ZnCl_2$ (0.0257 g), and sodium citrate dihydrate (1.334 g) were co-dissolved in deionized water (60 mL). Polyacrylamide (average molecular weight: 3×10^6 g mol^{-1}, 0.457 g) was subsequently slowly added into the solution under stirring. After the polymer was completely dissolved under continuous stirring, an aqueous hydrazine solution (2.7 mL, 80 wt%) was added under stirring within ~5 min. The resultant solution was subsequently sealed in a Teflon-lined stainless-steel autoclave with a capacity of 100 mL. The autoclave was kept in an isothermal oven at 200 °C for 12 h. The dark product was washed with ethanol and deionized water for several times and finally redispersed in ethanol (10 mL) for further use. For the synthesis of mesoporous Al_2O_3 microspheres loaded with the magnetic nanoparticles, 2.5 mL of the $Zn_{0.41}Fe_{0.59}Fe_2O_4$ nanoparticle solution obtained above was added into the precursor solution for the preparation of mesoporous Al_2O_3 microspheres. The product from aerosol spray was cleaned and separated with a magnet. The preparation of mesoporous Al_2O_3 microspheres co-

loaded with the magnetic nanoparticles and Au nanoparticles was similar, except that 2.86 g of the ethanolic $HAuCl_4$ solution mentioned above was supplied additionally.

Calcination. The collected products were thermally calcined at 400 °C for 4 h at a heating rate of 1 °C min^{-1}. For some products, the calcination temperature was changed to 500 °C or the calcination time was changed to 8 h in order to examine the effect of the calcination condition on the crystalline structure of the product. In particular, for Fe_2O_3, the amorphous Fe_2O_3 product was obtained by thermally treating the sprayed product at 400 °C for 4 h in air; the α-Fe_2O_3 product was produced by thermally treating the sprayed product at 500 °C for 4 h in air; the Fe_3O_4 product was made by thermally treating the α-Fe_2O_3 product at 400 °C for 4 h in a gas mixture of H_2 and N_2 at 5:95 (v/v); and the γ-Fe_2O_3 product was prepared by thermally treating the Fe_3O_4 product at 400 °C for 4 h in air.

Photocatalytic water oxidation under visible light. Photocatalytic water oxidation was performed with 50 mg of the catalyst in an aqueous $AgNO_3$ solution (50 mL, 20 mM) in a photocatalytic water-splitting system (Beijing Perfectlight Technology Co., Ltd.). The reaction was under visible light illumination with a Xe lamp (electrical power: 300 W) that was equipped with a UV-cutoff filter ($\lambda > 420$ nm). The system was connected to a vacuum pump and a gas chromatograph for online detection of oxygen evolution. A commercial α-Fe_2O_3 product (Aldrich, product number: 544884, nanopowder, particle size: < 50 nm) was also used as the photocatalyst for comparison. All of the catalyst samples were measured under the same conditions.

Gas sensing. The structure of the gas sensing device and the electrical measurement system were identical to those described in our recent work[31,32]. The sensing device was made of a

ceramic tube (Hanwei Electronics Co., Ltd., Henan Province, China). The length, outer diameter, and thickness of the tube were 5 mm, 2 mm and 0.2 mm, respectively. Two gold electrodes were printed circularly around the circumference of the tube and at the two ends, with a separation distance of 4 mm between them. For oxide coating on the tube, the mesoporous Fe_2O_3 sample (0.5 g) was first dispersed in ethanol (5 mL). The resultant solution was then deposited on the outer surface of the tube with a brush, followed by drying in air at 60 °C for 2 h and subsequently annealing at 350 °C for 2 h. The obtained oxide film was several ten micrometers in thickness. A small Pt coil was inserted into the tube as a resistance heater to control the temperature of the gas sensor. In order to improve the long-term stability, the sensor was kept at the working temperature for 48 h in advance before measurement. The sensing test was carried out in a home-designed chamber of 1 L in volume with an electrical measurement system (ART-2000A, Art Beijing Science and Technology Development Co., Ltd.) that was connected to the two gold electrodes through electrical feedthroughs. The sensing signal was measured as a voltage by use of a sampling resistor. A higher measured voltage corresponded to a larger current, and therefore a smaller resistance, when the oxide was exposed to a molecular vapor. A stationary-state gas distribution method was used for testing the response of the sensing device to a gas at different concentrations in dry air. Specifically, a gas to be detected, such as toluene vapor, was injected into the chamber with a syringe and mixed automatically with air in the chamber. The concentration of the gas vapor was estimated by taking into account the saturated vapor pressure of the gas at a given temperature, the injected vapor volume, and the chamber volume. With this system, the gas measurements down to 1 ppm can be performed, with a good degree of reproducibility. For comparison, the commercial α-Fe_2O_3 product mentioned above was also tested for gas sensing.

Supplementary references

1. El-Shereafy, E., Abousekkina, M. M., Mashaly, A. & El-Ashry, M. Mechanism of thermal decomposition and γ-pyrolysis of aluminum nitrate nonahydrate [$Al(NO_3)_3 \cdot 9H_2O$]. *J. Radioanal. Nucl. Chem.* **237**, 183–186 (1998).
2. Shaheen, W. M. & Selim, M. M. Thermal characterization and catalytic properties of the $ZnO–Co_3O_4/Al_2O_3$ system. *Int. J. Inorg. Mater.* **3**, 417–425 (2001).
3. Pacewska, B. & Keshr, M. Thermal transformations of aluminum nitrate hydrate. *Thermochim. Acta* **385**, 73–80 (2002).
4. Shanmugam, Y., Lin, F.-Y., Chang, T.-H. & Yeh, C.-T. Thermal decomposition of metal nitrates in air and hydrogen environments. *J. Phys. Chem. B* **107**, 1044–1047 (2003).
5. Melnikov, P., Nascimento, V. A., Arkhangelsky, I. V. & Zanoni Consolo, L. Z. Thermal decomposition mechanism of aluminum nitrate octahydrate and characterization of intermediate products by the technique of computerized modeling. *J. Thermal Anal. Calorim.* **111**, 543–548 (2013).
6. Stern, K. H. High temperature properties and decomposition of inorganic salts. Part 3. Nitrates and nitrites. *J. Phys. Chem. Ref. Data* **1**, 747–772 (1972).
7. Gubrynowicz, L. & Strömich, T. Study on the thermal decomposition of chromium(III) nitrate nonahydrate (CNN). *Thermochim. Acta* **115**, 137–151 (1987).
8. Małecki, A., Małecka, B., Gajerski, R. & Łabuś, S. Thermal decomposition of chromium(III) nitrate(V) nanohydrate. Different chromium oxides $CrO_{1.5+y}$ formation. *J. Thermal Anal. Calorim.* **72**, 135–144 (2003).
9. Melnikov, P., Nascimento, V. A., Arkhangelsky, I. V., Zanoni Consolo, L. Z. & de Oliveira, L. C. S. Thermolysis mechanism of chromium nitrate nonahydrate and

computerized modeling of intermediate products. *J. Thermal Anal. Calorim.* **114**, 1021–1027 (2013).

10. Gallagher, P. K., Schrey, F. & Prescott, B. The thermal decomposition of aqueous manganese (II) nitrate solution. *Thermochim. Acta* **2**, 405–412 (1971).
11. de Bruijn, T. J. W., de Ruiter, G. M. J., de Jong, W. A. & van den Berg, P. J. Thermal decomposition of aqueous manganese nitrate solutions and anhydrous manganese nitrate. Part 2. Heats of reaction. *Thermochim. Acta* **45**, 279–292 (1981).
12. Gadalla, A. M. & Yu, H.-F. Thermal decomposition of Fe(III) nitrate and its aerosol. *J. Mater. Res.* **5**, 1233–1236 (1990).
13. Elmasry, M. A. A., Gaber, A. & Khater, E. M. H. Thermal decomposition of Ni(II) and Fe(III) nitrates and their mixtures. *J. Thermal Anal.* **52**, 489–495 (1998).
14. Wieczorek-Ciurowa, K. & Kozak, A. J. The thermal decomposition of $Fe(NO_3)_3{\cdot}9H_2O$. *J. Thermal Anal. Calorim.* **58**, 647–651 (1999).
15. Petrov, K., Markov, L. & Rachev, P. Thermal decomposition of mixed magnesium(II)–cobalt(II) hydroxide nitrate crystals to $Mg_xCo_{3-x}O_4$ ($0 < x \leq 1$) spinels. *React. Sol.* **3**, 67–74 (1987).
16. Mansour, S. A. A. Spectrothermal studies on the decomposition course of cobalt oxysalts. *Mater. Chem. Phys.* **36**, 317–323 (1994).
17. Ehrhardt, C., Gjikaj, M. & Brockner, W. Thermal decomposition of cobalt nitrato compounds: preparation of anhydrous cobalt(II)nitrate and its characterization by infrared and Raman spectra. *Thermochim. Acta* **432**, 36–40 (2005).
18. Nikolic, R., Zec, S., Maksimovic, V. & Mentus, S. Physico-chemical characterization of thermal decomposition course in zinc nitrate–copper nitrate hexahydrates. *J. Thermal Anal. Calorim.* **86**, 423–428 (2006).

19. Berbenni, V., Milanese, C., Bruni, G. & Marini, A. Thermal decomposition of gallium nitrate hydrate $Ga(NO_3)_3 \cdot xH_2O$. *J. Thermal Anal. Calorim.* **82**, 401–407 (2005).

20. Melnikov, P., Nascimento, V. A. & Zanoni Consolo, L. Z. Thermal decomposition of gallium nitrate hydrate and modeling of thermolysis products. *J. Thermal Anal. Calorim.* **107**, 1117–1121 (2012).

21. Vratny, F., Kern, S. & Gugliotta, F. The thermal decomposition of cerium (III) nitrate hydrate. *J. Inorg. Nucl. Chem.* **17**, 281–285 (1961).

22. Strydom, C. A. & van Vuuren, C. P. J. The thermal decomposition of cerium(III) nitrate. *J. Thermal Anal.* **32**, 157–160 (1987).

23. Petrov, K., Lyubchova, A. & Markov, L. Synthesis and thermal decomposition of magnesium hydroxide nitrates. *Polyhedron* **8**, 1061–1067 (1989).

24. Ding, Z., Martens, W. & Frost, R. L. Thermal activation of copper nitrate. *J. Mater. Sci. Lett.* **21**, 1415–1417 (2002).

25. Meng, F. K. *et al.* Photocatalytic water oxidation by hematite/reduced graphene oxide composites. *ACS Catal.* **3**, 746–751 (2013).

26. Townsend, T. K., Sabio, E. M., Browning, N. D. & Osterloh, F. E. Photocatalytic water oxidation with suspended alpha-Fe_2O_3 particles-effects of nanoscaling. *Energy Environ. Sci.* **4**, 4270–4275 (2011).

27. Yin, S. L. *et al.* Synergistic contributions by decreasing overpotential and enhancing charge-transfer in α-Fe_2O_3/Mn_3O_4/graphene catalysts with heterostructures for photocatalytic water oxidation. *Phys. Chem. Chem. Phys.* **16**, 11289–11296 (2014).

28. Zhu, J. X. *et al.* Hierarchical hollow spheres composed of ultrathin Fe_2O_3 nanosheets for lithium storage and photocatalytic water oxidation. *Energy Environ. Sci.* **6**, 987–993 (2013).

29. Kakuta, S. & Abe, T. Photocatalysis for water oxidation by Fe_2O_3 nanoparticles embedded in clay compound: correlation between its polymorphs and their photocatalytic activities. *J. Mater. Sci.* **44**, 2890–2898 (2009).

30. Cheng, W., Tang, K. B. & Sheng, J. Highly water-soluble superparamagnetic ferrite colloidal spheres with tunable composition and size. *Chem. Eur. J.* **16**, 3608–3612 (2010).

31. Huang, J. R. *et al.* Size-controlled synthesis of porous $ZnSnO_3$ cubes and their gas-sensing and photocatalysis properties. *Sens. Actuators B* **171–172**, 572–579 (2012).

32. Geng, B. Y., Zhan, F. M., Fang, C. H. & Yu, N. A facile coordination compound precursor route to controlled synthesis of Co_3O_4 nanostructures and their room-temperature gas sensing properties. *J. Mater. Chem.* **18**, 4977–4984 (2008).
